# Correlates of Premenstrual Syndrome in Polish Adolescents—Results from POLKA 18 Youth-Led Cross-Sectional Study

**DOI:** 10.3390/jcm13237342

**Published:** 2024-12-02

**Authors:** Katarzyna Rylewicz, Michalina Anna Drejza, Grzegorz Łopiński, Ewa Majcherek, Joanna Barwińska, Małgorzata Mizgier, Katarzyna Plagens-Rotman, Magdalena Pisarska-Krawczyk, Witold Kędzia, Grażyna Jarząbek-Bielecka

**Affiliations:** 1 London School of Hygiene ∓ Tropical Medicine, London WC1E 7HT, UK; 2 Department of Obstetrics and Gynaecology, Cambridge University Hospitals NHS Foundation Trust, Cambridge CB2 0QQ, UK; michalina.drejza@gmail.com; 3 Samodzielny Publiczny Zakład Opieki Zdrowotnej, 08-110 Siedlce, Poland; gregory.lopinski@gmail.com; 4 University Clinical Hospital in Poznan, 60-355 Poznan, Poland; ewaa.majcherek@gmail.com; 5 Murcki Hospital, 40-749 Katowice, Poland; jbarwinska21@gmail.com; 6 Department of Sports Dietetics, Faculty of Health Sciences, Poznan University of Physical Education, Królowej Jadwigi 27/39, 61-871 Poznan, Poland; mizgier@awf.poznan.pl; 7 Department of Gynaecology, Division of Gynaecology, Poznan University of Medical Sciences, 61-701 Poznan, Poland; plagens.rotman@gmail.com (K.P.-R.); witold.kedzia@poczta.fm (W.K.); graja@ump.edu.pl (G.J.-B.); 8 Department of Nursing, The President Stanislaw Wojciechowski Calisia University, 62-800 Kalisz, Poland; magmp@op.pl

**Keywords:** premenstrual syndrome, menstrual health, adolescent girls, Poland, mental health, risk behaviours, women’s health

## Abstract

**Objectives:** This study aims to evaluate the prevalence of premenstrual syndrome (PMS) among Polish adolescents and explore its associations with mental health outcomes, lifestyle factors, and risk behaviours. Additionally, it seeks to examine the impact of PMS on quality of life, contributing to the foundation for initiatives that enhance adolescent menstrual health. **Methods:** This research is part of the POLKA 18 study, a youth-led cross-sectional survey conducted between April and December 2019. Final-year high school students were surveyed using paper-based, self-reported questionnaires. Statistical analysis was performed using the R programming language in RStudio, with a significance threshold of *p*-value < 0.05. **Results:** A total of 1545 valid responses were analysed. PMS was reported by 33.9% of respondents, with over 80% experiencing premenstrual symptoms. Adolescents with negative mental health outcomes, such as self-harm or suicidal ideation, were significantly more likely to report PMS (*p* ≤ 0.001). PMS was also strongly associated with anxiety and panic attacks (*p* < 0.001). Risk behaviours, including smoking (*p* = 0.006), illicit substance use (*p* < 0.01), and increased alcohol consumption in the past 30 days (*p* < 0.001), were more prevalent among adolescents with PMS. Furthermore, respondents with PMS reported poorer school performance (*p* = 0.002), higher stress levels (*p* < 0.001), and a more negative perception of their overall health (*p* < 0.001) compared to peers without PMS. **Conclusions:** PMS is significantly associated with mental health issues, engagement in risky behaviours, higher stress, and diminished self-perceived health and academic performance. These findings highlight the need for further research and the development of targeted interventions to improve adolescent menstrual health.

## 1. Introduction

Premenstrual syndrome (PMS) is a disorder that occurs during the luteal phase of the menstrual cycle, before the onset of menstruation, and causes a range of somatic and psychological symptoms. Although the aetiology of PMS remains unclear, it is thought to be caused by fluctuating levels of oestrogen and progesterone and their imbalance, as well as genetic and neurobiological factors [1,2]. PMS affects approximately 20–30% of menstruating women, with up to 80% reporting physical and emotional changes. Meanwhile, 2.5 to 5% of women experience significant impairment in their daily functioning [3].

The Royal College of Obstetricians and Gynaecologists (RCOG) has described diagnostic criteria for PMS. To make a diagnosis, patients should record premenstrual symptoms and their impact on daily life for two consecutive menstrual cycles. The diagnostic symptoms of PMS can be divided into two groups. The first group includes psychological symptoms such as depression, anxiety, irritability, mood swings, or loss of confidence. The second group comprises physical symptoms including mastalgia, abdominal bloating, or headaches [4].

Premenstrual dysphoric disorder (PMDD) is a severe manifestation of PMS with a prevalence of 3–8% [2]. In PMDD, symptoms are severe to the point of interfering with work, social situations, and family relationships [5]. It is important to note that PMS can also cause social withdrawal, decreased academic performance [6], and reduced quality of life [7], as outlined by the RCOG diagnostic criteria described above.

Research indicates a significant association between PMS and self-harm [8,9]. A systematic review has shown that menstrual fluctuations in anxiety symptoms are present in various diagnoses, particularly panic disorder [10]. A recent meta-analysis has found that women with either PMS or PMDD have a higher risk of suicidal behaviour [11].

PMS has been associated with risk behaviours such as smoking cigarettes, alcohol consumption, and illicit substance use. A meta-analysis found a link between smoking and a higher risk of recurrent PMS [12]. Additionally, it was suggested that smoking may be used as a coping mechanism to manage PMS-induced dysphoria [13,14]. As a result, it may worsen symptoms, as current and former smokers report higher levels of negative menstrual symptoms; however, the correlation remains unclear [15]. Similarly to smoking, another meta-analysis indicated that alcohol consumption is more prevalent among patients with PMS; however, the data are insufficient to imply causation [16]. Furthermore, a prospective cohort study has established a correlation between the use of illicit drugs and the risk of recurrent PMS [17]. This emphasises the intricate associations among health risk behaviours, mental wellbeing, and PMS, highlighting the need for further investigation into this topic.

PMS is believed to be influenced by lifestyle factors such as diet, exercise, sleep, and stress levels [18,19,20,21,22]. The relationship between PMS and body mass index (BMI) is inconclusive, with conflicting evidence from various studies [23,24]. However, exercise has been suggested as a potential treatment for PMS [25].

Previous studies have shown that PMS negatively affects school performance in adolescents [26,27,28,29]. Additionally, some studies have suggested that stress may increase the incidence of PMS among adolescent girls [30,31,32]. Moreover, PMS has been found to have a negative impact on health-related quality of life [33,34,35].

As there have been few studies on PMS among Polish adolescents, this research aims to evaluate its prevalence and associations with mental health, lifestyle factors, and risk behaviours. Additionally, it aims to examine the effects of PMS on the quality of life of Polish adolescents and establish the foundation for the creation and implementation of initiatives aimed at improving adolescent health.

## 2. Materials and Methods

This paper presents results from the POLKA 18 project, which was a youth-led cross-sectional study aimed at the assessment of knowledge, attitudes, and practices of Polish adolescents in regard to their health and healthcare, with particular emphasis on sexual and reproductive health.

The research consisted of two phases, namely the pilot and phase II, with both relying on bespoke paper-based questionnaires self-reported by the respondents. The survey questionnaires were disseminated by medical students, who were members of the Association of Medical Students IFMSA-Poland, in high schools and vocational schools that agreed to participate in this study. The selection of schools was based on prior collaboration with IFMSA-Poland, while some schools were newly recruited by research assistants. Only students in their final year of high school and penultimate year of vocational school were surveyed to ensure that they were of legal age (18 or older) to participate in this study without requiring parental consent. The majority of adolescents who took part in this study were between the ages of 18 and 19. However, some of the participants from vocational schools were above the age of 19. The questionnaires were completed at the time of peer education classes facilitated by IFMSA-Poland representatives. The questionnaire’s cover page contained information for the participants about the topic of this study, guaranteed anonymity, a request not to record any personal data, and notification regarding their right to refuse to participate in this study. For the purposes of this analysis, all data used in this study were retrieved from the POLKA 18 questionnaire.

The questionnaires were distributed in six Polish regions (voivodeships): Mazowieckie, Śląskie, Wielkopolskie, Lubelskie, Pomorskie, and Zachodniopomorskie—only in phase II.

Respondents were asked whether they had ever been diagnosed with PMS, and their answers were used to define the groups for comparison. While a question about PMS symptoms was included as an additional data point, it was not considered when comparing groups for mental health and risk behaviour outcomes.

The obtained data were cleaned by scanning the records for missing and incorrect entries for each variable. For intergroup comparisons of qualitative variables, the Chi2–Pearson test was used. For quantitative variables with a distribution significantly different from the normal distribution, the Wilcoxon test was used. Kendall’s coefficient was used to assess correlations between variables. The analysis was performed using the R language in the RStudio environment Posit team (2024). A *p*-value < 0.05 was considered significant.

All student participants provided voluntary informed consent. The ethics clearance was obtained from the Ethics Committee of Poznan University of Medical Sciences (document date: 13 December 2018, signature number: KB 540/24). Data handling adhered to the ethical principles of the Declaration of Helsinki, ensuring anonymity throughout the study process.

## 3. Results

### 3.1. General Characteristics of the Study Group

For the final analysis of this research, a total of 1545 questionnaires from the POLKA 18 study were included. The criterion for inclusion in this study was a positive answer to the following question: “Have you started menstruating yet?”. The general characteristics of the sample can be found below, in Table 1.

### 3.2. Premenstrual Syndrome

#### 3.2.1. Symptoms

Premenstrual syndrome was reported by over one third of the respondents (33.9%). However, when questioned about specific symptoms occurring before menstruation in the last year, the following were declared: 88.9% mood swings, 86.5% nervousness, 85.9% abdominal pain, 77.1% tearfulness/sadness, 69.6% increased appetite, 69.2% anger issues, 61.0% increased breast tension, 60.9% impaired concentration, 43.2% dizziness, and 30.0% nausea/vomiting. Table 2 presents a summary of the symptoms reported, including the number of respondents and corresponding percentages.

#### 3.2.2. Association with Mental Health Outcomes

Individuals with PMS were significantly more likely to report negative mental health outcomes, including anxiety, panic attacks, suicidal ideation, and self-harm. Among those with PMS, 58.2% experienced panic attacks in the past year, whereas only 40.1% of individuals without PMS reported the same (*p* < 0.001). Similarly, anxiety in the past year was reported by 70.3% of adolescents with PMS, in contrast to 52.6% of those without PMS (*p* < 0.001).

Moreover, suicidal ideation was noted in 28.5% of individuals with PMS, a notably higher percentage than the 19.6% observed in those without PMS (*p* < 0.001). Self-harm was also more prevalent, affecting 14.4% of individuals with PMS, as opposed to 8.6% of those without the condition (*p* = 0.001).

When questioned about their level of happiness (assessed with the use of the Likert scale), 47.4% of individuals diagnosed with PMS answered positively (yes or definitely yes), in contrast to 59.7% of those without a PMS diagnosis.

A summary of these responses and their corresponding *p*-values are shown in Table 3 below.

#### 3.2.3. Association with Health Risk Behaviours

We have asked our respondents about several risk behaviours including smoking cigarettes, drinking alcohol, and use of illicit drugs. Our results with numbers and percentages of responses, characterised by PMS presence, together with *p*-values, can be found below in Table 4.

A higher proportion of those diagnosed with PMS reported ever having smoked a cigarette compared to those without PMS—71.7% vs. 64.3% (*p* < 0.006). Nevertheless, no statistically significant association was observed between PMS and the quantity of cigarettes consumed in the previous 30 days (*p* = 0.194).

There was a significant link (*p* = 0.005) between the consumption of alcoholic drinks within the past 30 days and the prevalence of PMS. Furthermore, a comparable linkage held true for experiences of intoxication, subsequent regret, and memory loss (all *p* < 0.001).

There was also a statistically significant association identified between individuals who have ever used marijuana (*p* < 0.001) or other drugs (*p* = 0.019) and the prevalence of PMS.

#### 3.2.4. Association with School Health and Lifestyle Factors

We have asked the participants about their performance at school, levels of stress, hours of sleep per night on a school day, physical activity, and general perception of overall health. The results, characterised by the presence of PMS, are summarised in Table 5 below.

There was a significant discrepancy in the perception of school performance between individuals with PMS and those without PMS (*p* = 0.002). Those experiencing PMS tended to view their performance as average, below average, bad or very bad more frequently.

Similarly, the stress levels reported by individuals with PMS were significantly higher than those reported by adolescents without a PMS diagnosis (*p* < 0.001).

Additionally individuals without a diagnosis of PMS demonstrated a more positive perception of overall health (good or very good health) compared to those with PMS diagnosis (*p* < 0.001).

However, there was no significant association between PMS diagnosis and the level of physical activity (*p* = 0.074) or average hours of sleep on a school day (*p* = 0.065).

## 4. Discussion

### 4.1. Main Findings

One third of respondents reported experiencing premenstrual syndrome, with over 80% of menstruating adolescents reporting related symptoms such as mood swings, nervousness, and abdominal pain, which is consistent with other studies reporting a similar percentage [3]. This underscores the high prevalence of PMS among Polish adolescents and suggests the possibility that some individuals may not recognize PMS symptoms as concerning, may have limited awareness of the condition, or may face barriers to seeking medical care, leading to underdiagnosis.

Individuals with PMS reported worse mental health outcomes, indicating a statistically significant difference. Previous research has demonstrated a correlation between premenstrual syndrome and elevated suicidal rates, depression, and anxiety [8,9,10,11]. Further investigation is required to establish the causal mechanism, including an exploration of the potential impact of stress on premenstrual syndrome.

This study found that adolescents with PMS were using illicit drugs more often, which is in accordance with previous studies [17]. While marijuana may provide some benefits for premenstrual syndrome negative symptoms, its use is contraindicated in adolescents due to negative effects on sexual and reproductive health, mental health, and brain development [36,37]. It was also found to be associated with higher PMS prevalence in our study. Similarly, our study found a strong association between alcohol consumption and higher PMS prevalence, taking into account both the number of alcoholic drinks consumed in the last month and experience of ever being drunk. The incidence of PMS was found to be higher among those who have smoked cigarettes. Overall, these findings suggest that PMS is not only a physical health concern but may also be connected to broader behavioural and mental health challenges in adolescents. This highlights the need for targeted interventions to address both PMS management and the associated risky behaviours.

Moreover, Polish adolescents with PMS reported worse school performance and higher stress levels compared to their healthy counterparts. These findings are consistent with previous studies [26,27,28,29,30,31,32]. Additionally, the participants’ self-perceived health was worse in the group with diagnosed PMS. As adolescents with premenstrual syndrome (PMS) often have a lower health-related quality of life than the general population, it may be beneficial to establish national clinical guidelines for PMS management. Additionally, improving health literacy through school programmes could enhance their understanding of their own bodies and menstrual health, and encourage them to seek specialist care if they experience negative symptoms.

Our study found no association between levels of exercise or hours of sleep and the incidence of PMS, despite previous studies suggesting such associations [25]. It shows that further research is required to comprehend the relationship between PMS and lifestyle and to establish causal mechanisms.

### 4.2. Strengths and Limitations

The POLKA 18 study, which was used as a source of data for this analysis, has a relatively large sample size which is representative of the Polish population. Many population-based studies among adolescents are single-centre studies, which survey young people in just one school or town. This study considers the geographical distribution and representation of different regions, allowing for a comprehensive analysis of the Polish adolescent population. However, our study was constrained by the legal age of consent, which is 18 years old. This limitation restricts our ability to include adolescents in the broader definition, as we only examined individuals aged 18 or over. Moreover, the data cannot be extrapolated to younger adolescents as patterns of engagement in risky behaviours, such as smoking, alcohol consumption, and drug use change with advancing age.

In this study, participants were categorised as having PMS based on a positive response to the question regarding a formal PMS diagnosis. However, the prevalence of reported negative symptoms over the past year was significantly higher, highlighting a limitation in our approach. Some respondents may have experienced PMS symptoms without receiving a formal diagnosis, leading to a potential underestimation of the true prevalence of PMS. Further research is needed to establish closer links and correlations between self-reported symptoms and formal diagnostic criteria to improve the accuracy of prevalence estimates.

Additionally, when comparing adolescents with and without PMS, it is important to consider how negative mental health outcomes, such as anxiety and depression, independently contribute to the observed associations and may influence the results. Previous research has demonstrated that both anxiety and depression are significant predictors of risky behaviours [15]. Thus, the relationship between PMS and these behaviours may be compounded by underlying mental health issues. This highlights the need for further analysis to disentangle the direct effects of PMS from the broader impact of mental health on risky behaviours.

The team encountered difficulties in reaching more students as some schools declined to participate in the survey because it covered sexual and reproductive health issues. Investigating topics like menstrual health, sexual behaviours, and contraceptive use among adolescents is often met with public resistance in Poland. To mitigate this issue, convenience sampling was used in some cases based on previous cooperation with schools, which may have introduced some bias.

The questionnaires were self-reported, enabling us to explore sensitive topics such as smoking, alcohol consumption, sexual, and contraceptive behaviours, and obtain more candid responses. However, due to that, we had limited ability to verify the accuracy of the responses and to account for external factors that may have influenced them.

In this study, we did not inquire about the participants’ use of medications for premenstrual symptoms, including SSRIs and hormonal contraceptives, which are approved treatment methods [4]. This could be considered a limitation as we did not investigate the impact of these medications on the reported symptoms and behaviours.

## 5. Conclusions

In summary, our research indicates a significant association between PMS symptoms and mental health problems, particularly suicidal ideations, self-harm, anxiety, and panic attacks. Adolescents with PMS are more likely to engage in high-risk health behaviours, such as smoking cigarettes or marijuana, taking illicit drugs, or drinking alcohol, compared to their healthy counterparts. They also experience higher levels of stress, have poorer academic performance, and perceive their overall health to be worse. We found no associations between PMS and sleep patterns, or exercise. Further research is needed to investigate the influence of lifestyle on PMS. There is a strong need for better school-based education programmes to improve adolescents’ health literacy, including that pertaining to menstrual health, in order to increase their understanding of their own bodies and to encourage them to seek specialist care if they experience negative symptoms.

## Figures and Tables

**Table 1:** General characteristics of the sample (*n* = 1545) (%).

Age	
18	1216 (78.7%)
19	250 (16.2%)
Above 19	79 (5.1%)
Gender	
Female	1538 (99.5%)
Other	7 (0.5%)
Region of Poland	
Mazowieckie	414 (26.8%)
Śląskie	294 (19%)
Wielkopolskie	360 (23.3%)
Pomorskie and Zachodniopomorskie	292 (18.9%)
Lubelskie	184 (11.9%)
Residence	
City over 100,000	505 (32.8%)
City under 100,000	485 (31.5%)
Rural area	548 (35.6%)

**Table 2:** Premenstrual symptoms reported by adolescents—*n* (%).

Symptoms Occurring Before Menstruation in the Last Year—*n* (%)	
Mood swings	1333 (88.9)
Nervousness	1295 (86.5)
Abdominal pain	1289 (85.9)
Tearfulness/sadness	1153 (77.1)
Increased appetite	1041 (69.6)
Anger issues	1029 (69.2)
Increased breast tension	909 (61.0)
Impaired concentration	904 (60.9)
Dizziness	641 (43.2)
Nausea/vomiting	444 (30.0)

**Table 3:** Association of PMS with mental health, sample characterised by presence of PMS [=x (%)].

PMS	Yes	No	*p*-Value
N (%)	494 (33.9)	964 (66.1)	
In the past year, have you ever had a panic attack? N (%)			
Yes	287 (58.2)	384 (40.1)	<0.001
No	206 (41.8)	574 (59.9)	
In the past year, have you ever felt anxious? N (%)			
Yes	345 (70.3)	504 (52.6)	<0.001
No	146 (29.7)	454 (47.4)	
Have you thought about suicide in the past year? N (%)			
Yes	140 (28.5)	187 (19.6)	<0.001
No	351 (71.5)	767 (80.4)	
In the past year, have you self-harmed yourself? N (%)			
Yes	71 (14.4)	82 (8.6)	0.001
No	422 (85.6)	875 (91.4)	
Do you feel happy? N (%)			
Definitely yes	50 (10.2)	154 (16.2)	<0.001
Yes	182 (37.2)	415 (43.5)	
Don’t know	187 (38.2)	301 (31.6)	
No	56 (11.5)	62 (6.5)	
Definitely no	14 (2.9)	21 (2.2)	

**Table 4:** Risk behaviours and PMS diagnosis, characterised by the presence of PMS [=x (%)].

PMS	Yes	No	*p*-Value
*n*	494	964	
Have you ever smoked cigarettes? N (%)			
Yes	354 (71.7)	617 (64.3)	0.006
No	140 (28.3)	342 (35.7)	
In the last 30 days, on how many days have you had a cigarette? N (%)			
0 days	266 (54.5)	589 (61.9)	0.194
1–2 days	62 (12.7)	108 (11.4)	
3–5 days	33 (6.8)	52 (5.5)	
6–9 days	24 (4.9)	34 (3.6)	
10–19 days	28 (5.7)	38 (4.0)	
20–29 days	19 (3.9)	32 (3.4)	
Every day	56 (11.5)	98 (10.3)	
In the last 30 days, on how many days did you drink at least one alcoholic drink? N (%)			
0 days	78 (15.8)	214 (22.3)	0.005
1–2 days	147 (29.8)	316 (33.0)	
3–5 days	147 (29.8)	208 (21.7)	
6–9 days	74 (15.0)	136 (14.2)	
10–19 days	36 (7.3)	61 (6.4)	
20–29 days	9 (1.8)	13 (1.4)	
Every day	3 (0.6)	11 (1.1)	
Have you ever been drunk? N (%)			
Yes	410 (83.3)	707 (73.6)	<0.001
No	82 (16.7)	254 (26.4)	
Have you ever regretted something you did under the influence of alcohol? N (%)			
Yes	247 (50.3)	365 (38.1)	<0.001
No	244 (49.7)	592 (61.9)	
Have you ever forgotten what you were doing while under the influence of alcohol? N (%)			
Yes	222 (44.9)	332 (34.7)	<0.001
No	272 (55.1)	626 (65.3)	
Have you ever tried marijuana? N (%)			
Yes	216 (43.7)	313 (32.5)	<0.001
No	278 (56.3)	649 (67.5)	
Have you ever tried drugs other than marijuana (e.g., LSD, cocaine, ecstasy, hashish, heroin)? N (%)			
Yes	54 (11.0)	69 (7.2)	0.019
No	439 (89.0)	893 (92.8)	

**Table 5:** Risk behaviours and PMS diagnosis, characterised by the presence of PMS [=x (%)].

PMS	Yes	No	*p*-Value
*n*	494	964	
Over the past academic year, how do you feel about your performance in school? N (%)			
Very well	45 (9.2)	122 (12.7)	0.002
Above average	95 (19.3)	260 (27.2)	
Average	271 (55.2)	456 (47.6)	
Below average	40 (8.1)	55 (5.7)	
Bad	30 (6.1)	47 (4.9)	
Very bad	10 (2.0)	17 (1.8)	
On a scale of 1 (very low)–10 (very high), how would you rate your stress level? N (%)			
1	2 (0.4)	17 (1.8)	<0.001
2	7 (1.4)	39 (4.1)	
3	21 (4.3)	66 (6.9)	
4	30 (6.2)	102 (10.7)	
5	49 (10.1)	149 (15.7)	
6	41 (8.5)	106 (11.2)	
7	70 (14.4)	132 (13.9)	
8	114 (23.5)	159 (16.7)	
9	66 (13.6)	86 (9.1)	
10	85 (17.5)	94 (9.9)	
During a typical school day, how many hours do you sleep per night? N (%)			
4 or less	30 (6.2)	56 (5.9)	0.065
5	74 (15.2)	129 (13.6)	
6	161 (33.1)	251 (26.4)	
7	142 (29.2)	331 (34.8)	
8	65 (13.3)	149 (15.7)	
9 or more	15 (3.1)	36 (3.8)	
During the last week, on how many days were you physically active for at least 60 min a day? N (%)			
0	100 (20.3)	148 (15.5)	0.074
1	95 (19.3)	144 (15.1)	
2	96 (19.5)	197 (20.7)	
3	94 (19.1)	215 (22.6)	
4	46 (9.3)	111 (11.6)	
5	30 (6.1)	74 (7.8)	
6	12 (2.4)	22 (2.3)	
7	19 (3.9)	42 (4.4)	
How would you rate your overall health? N (%)			
very bad	4 (0.8)	8 (0.8)	<0.001
bad	44 (8.9)	64 (6.7)	
average	159 (32.3)	230 (24.1)	
good	229 (46.5)	478 (50.0)	
very good	56 (11.4)	176 (18.4)	

## Data Availability

The original contributions presented in the study are included in the article, further inquiries can be directed to the corresponding author.
